# Assessment of the tuberculosis case-finding and prevention cascade among people living with HIV in Zambia – 2018: a cross-sectional cluster survey

**DOI:** 10.1186/s12889-021-10929-z

**Published:** 2021-05-04

**Authors:** Michael Melgar, Ray W. Shiraishi, Clifford Tende, Sydney Mwanza, Joyce Mulenga, Shepherd Khondowe, David Mwakazanga, Kelvin Kapungu, Mathias Tembo, Amos Nota, Patrick Lungu, Brittany Moore, Laura J. Podewils

**Affiliations:** 1 Division of Global HIV and Tuberculosis, Centers for Disease Control and Prevention, 1600 Clifton Road, Mailstop US1-1, Atlanta, GA 30030 USA; 2 Tropical Diseases Research Centre, Ndola, Zambia; 3 National Tuberculosis and Leprosy Control Programme, Ministry of Health, Lusaka, Zambia

**Keywords:** Tuberculosis, Screening, Preventive treatment, Uptake, Cascade of care, Co-infection

## Abstract

**Background:**

The Ministry of Health Zambia recommends tuberculosis preventive treatment (TPT) with 6 months daily isoniazid for all people living with human immunodeficiency virus (HIV) after ruling out active tuberculosis disease. We sought to estimate the percentage of people living with HIV who progress through each stage of the tuberculosis case-finding and prevention cascade in two provinces with the highest tuberculosis burden in Zambia.

**Methods:**

In this cross-sectional survey, we used a two-stage cluster sampling method. We sampled 12 healthcare facilities with probability proportional to size. Patient volume determined facility cluster size. During October 2018, from each facility we systematically sampled medical records of adults and children living with HIV. Our primary outcome of interest was TPT initiation rate among eligible people living with HIV, weighted for complex survey design. The Rao-Scott adjusted chi-square test was used to test for differences in TPT initiation rate and other indicators from the tuberculosis prevention cascade by age group and province of residence. Additionally, we conducted semi-structured interviews with healthcare workers at each facility to assess TPT knowledge and identify challenges to its implementation.

**Results:**

We sampled 482 records of people living with HIV (including 128 children living with HIV). Excluding two people diagnosed with tuberculosis disease before enrollment in HIV care, 93.4% underwent tuberculosis symptom screening. Of those, 4.7% were diagnosed with tuberculosis disease and 95.3% were TPT-eligible, of whom 24.7% initiated TPT. TPT initiation was lower among eligible children (7.7%) compared with adults (25.2%, *p* = 0.03) and Copperbelt residents (3.1%) compared with Lusaka residents (35.8%, *p* < 0.01). TPT completion rate was 38.4% among people living with HIV who initiated the 6-month course. Among interviewed healthcare workers, 58.3% (unweighted) incorrectly relayed the number of symptoms needed for a positive tuberculosis symptom screen, 83.3% (unweighted) reported insufficient isoniazid stockpile for completion at the time of TPT initiation, and only 27.3% (unweighted) reported receiving TPT-specific training.

**Conclusions:**

TPT uptake among people living with HIV in Zambia is challenged by inconsistent tuberculosis screening, lack of TPT training for healthcare workers, and supply chain inefficiencies. Addressing these barriers may increase TPT initiations and improve outcomes among people living with HIV.

**Supplementary Information:**

The online version contains supplementary material available at 10.1186/s12889-021-10929-z.

## Introduction

Tuberculosis (TB) is the leading cause of death among people living with human immunodeficiency virus (PLHIV), estimated to account for nearly one third of all deaths [1]. Antiretroviral therapy (ART) reduces TB incidence [2]. Increases in ART coverage over the last decade have prevented millions of TB cases and deaths [3, 4], but TB-related mortality remains higher among PLHIV on ART than among HIV-uninfected persons [1]. TB preventive treatment (TPT) also decreases TB incidence and mortality among PLHIV [5, 6], with an effect independent from that of ART [7]. The World Health Organization (WHO) recommends TPT for PLHIV without active TB disease, including children living with HIV (CLHIV) aged ≥12 months and pregnant and breastfeeding women with HIV, with 6 months of daily isoniazid (with pyridoxine supplementation to prevent peripheral neuropathy) or an equivalent rifamycin-based regimen [8]. A negative clinical screen for cough, fever, night sweats, and weight loss (among children: cough, fever, poor weight gain, and contact with a patient with TB disease) is recommended for case finding and to rule out active TB disease for TPT eligibility [8, 9]. In resource-limited settings, TPT should not be delayed for testing for latent TB infection (LTBI) with tuberculin skin testing or interferon gamma release assay [8].

Zambia has among the world’s highest burdens of TB and HIV. A 2013–2014 national prevalence survey revealed an estimated prevalence of bacteriologically confirmed TB disease of 638/100,000 among adults and 1726/100,000 among adult PLHIV [10]. In 2019, an estimated 59,000 people were diagnosed with TB disease, of whom nearly 50% were coinfected with HIV [1]. An estimated 40% of cases in Zambia were undiagnosed or untreated [1], suggesting high rates of community transmission and prevalent LTBI. There were 15,400 TB deaths, 9500 of which were among PLHIV [1]. HIV prevalence was estimated to be 11.4% among adults aged 15–49 years [11]. In 2019, there were 1.2 million PLHIV in Zambia [12]. Of those, 1,064,000 (89%) were on ART [12] including 939,790 who received treatment through support from the United States President’s Emergency Plan for AIDS Relief (PEPFAR) [13].

Since 2016, the Ministry of Health Zambia (MOH), in partnership with PEPFAR, has incorporated TPT with 6 months of isoniazid monotherapy with pyridoxine supplementation into the standard package of HIV care across the country. In 2018, as part of a plan to scale up the national TPT program, the MOH published updated HIV management guidelines prioritizing TPT among newly diagnosed PLHIV starting ART [14]. However, in resource-limited settings, numerous supply chain [15, 16], policy [16], logistical [17], social [18], and clinical [19, 20] barriers have been reported to impede programmatic TPT scale-up. In Zambia, the WHO reported that in 2017, only 18% of PLHIV newly enrolled in ART were initiating TPT [21]. The extent to which PLHIV are screened for TB, TPT initiation rates among those who are eligible, and completion rates among those who initiate TPT remain unclear.

To evaluate the TB case-finding and prevention cascade in Zambia, we used a stratified, two-stage cluster sampling design to retrospectively evaluate quantitative clinical and surveillance data, as well as qualitative interview data from healthcare professionals working at select facilities offering ART through PEPFAR support in Lusaka and Copperbelt Provinces, the two Zambian provinces with the highest TB and HIV burdens [22].

## Methods

### Assessment of the TB case finding and prevention cascade

We conducted a stratified, two-stage cluster survey (Supplemental Figure). The sampling frame included all PEPFAR-affiliated ART facilities in Lusaka (*N* = 156) and Copperbelt (*N* = 175) Provinces. In the first stage, 12 ART facilities were selected using probability-proportional-to-size sampling, where facility size was based on the number of PLHIV newly started on ART during the most recent United States government fiscal year for which PEPFAR programmatic data was available (October 1, 2016 to September 30, 2017; total across Lusaka and Copperbelt Provinces: 74,136). In the second stage, up to 30 adults and 30 children with ART initiation dates from January 1–June 30, 2018 were selected separately from each facility using systematic sampling from facility paper-based ART registers. The sampling interval was determined by the total number of adults and children reported in the ART facility register during the six-month study period (i.e., if N adults [or children] were reported, sampling interval was N/30). If fewer than 30 adults or children had initiated ART during the six-month period, we used a take-all approach.

For sample size calculations, we calculated the 95% confidence interval (CI) width (i.e., Wilson score method with continuity correction) that would be achieved for expected sample sizes of 360 adults and 200 children, assuming 52% were screened for TB, 98% uptake of TPT among eligible PLHIV, and 23% completion among PLHIV who started TPT, which were based on existing programmatic data. To account for the complex survey design, the calculations were performed for samples sizes of 360, 240, 180 and 120 for adults and 200, 133, 100, and 67 for pediatric patients, corresponding to design effects of 1.0, 1.5, 2.0, and 3.0, respectively. For our purposes, the sample size calculations suggested that expected PLHIV screened for TB and uptake of TPT among PLHIV would generally achieve acceptable levels of precision (i.e., ±10%). Sample size calculations were performed using PASS 15.0.4 [23].

We conducted site visits to each of the 12 facilities during October 2018. For each sampled PLHIV, we reviewed the medical record (paper-based and electronic) and the laboratory TB register to determine whether the individual had a diagnosis of active TB disease prior to ART initiation, whether s/he underwent TB symptom screening per WHO guidelines (four symptoms: cough, fever, night sweats, weight loss), and whether active TB disease was diagnosed at the time of ART initiation. A new diagnosis of active TB disease was assigned if: 1) the laboratory register contained a positive microscopy result for acid-fast bacilli, a positive mycobacterial culture, or a positive Gene Xpert MTB/RIF (Cepheid, Sunnyvale CA) result on any clinical specimen; or 2) the medical record contained a statement by the clinician diagnosing active TB disease or contained documentation of a prescription for multi-drug, anti-TB treatment regimen. We assigned the date of diagnosis as the earliest date of laboratory confirmation, treatment start, or documentation of diagnosis in the medical record.

Conversely, we determined the individual was TPT-eligible if s/he had a negative symptom screen and was not diagnosed with active TB disease, or if s/he had a positive symptom screen but was not diagnosed with active TB disease after at least one follow-up appointment. Other relative and absolute contraindications to isoniazid, such as previous hypersensitivity reaction to isoniazid, chronic liver disease, or daily alcohol use were omitted from TPT eligibility criteria because they were not systematically recorded. If the individual was TPT-eligible, we reviewed the medical record and the pharmacy register to assess whether s/he initiated TPT, and if so, whether the individual either completed TPT or was still receiving TPT at the time of data abstraction. Data were abstracted using a paper form and then entered into a structured electronic database using Epi Info version 7.2.2.6 (Centers for Disease Control and Prevention, Atlanta, GA).

We calculated population-level estimates of eight key indicators important to the programmatic scale-up of TPT in Zambia. These included:

1. The percentage of new-to-ART PLHIV who underwent TB symptom screening,
2. The percentage of screened PLHIV who had a positive TB symptom screen (> 1 of 4 symptoms),
3. The percentage of PLHIV with a positive TB symptom screen who underwent microbiological evaluation for TB (microscopy, mycobacterial culture, or nucleic acid amplification test on a clinical specimen),
4. The percentage of screened PLHIV who were diagnosed with active TB disease,
5. The percentage of screened PLHIV who were TPT-eligible,
6. The percentage of TPT-eligible PLHIV who initiated TPT,
7. The percentage of PLHIV who completed TPT among those due for completion (having started TPT > 212 days [7 months] prior to data abstraction), and
8. Percentage who started and completed TPT among TPT-eligible PLHIV who started ART > 212 days prior to data abstraction.

All analyses were controlled for the complex design of the survey (i.e., clustering, stratification, and weighting). Indicators were estimated overall and stratified by age category and province. We evaluated for differences in indicators across demographic and geographic strata using the first-order, Rao-Scott chi-square statistic. Data were analyzed using R version 3.4.4 (The R Foundation for Statistical Computing, Vienna, Austria). Survey analyses were performed using the survey R package. Domain analyses were performed on subpopulations.

### Healthcare worker interviews

To assess knowledge of TB case-finding and TPT guidelines and identify perceived barriers to TPT implementation, we conducted semi-structured informational interviews with at least one healthcare worker (HCW) at each sampled ART facility. Target HCWs for interviews were those who made clinical management decisions for patients on ART and were able to prescribe TPT. These HCWs included physicians, clinical officers, and nurses. If target HCWs were unavailable or refused, we interviewed any available HCW involved in provision of TPT to PLHIV at the facility. Follow up questions to HCW responses were permitted and any information volunteered by HCWs was also recorded.

Basic occupational characteristics of each HCW were recorded and summarized. Open-ended question responses were thematically coded following review and group discussion. We calculated unweighted frequencies of responses.

## Results

### Assessment of the TB case finding and prevention cascade

We sampled 482 PLHIV (354 adults, 128 CLHIV) initiated on ART during January 1–June 30, 2018 across 12 ART facilities (Table 1). Eight sampled ART facilities were in Lusaka Province and four were in Copperbelt Province. One was a government hospital, eight were government clinics or health centers, and three were private hospitals or clinics. Seven had on-site TB laboratory services including acid-fast bacilli smear microscopy and mycobacterial culture. Two sampled PLHIV had a diagnosis of active TB disease prior to ART initiation and were excluded, leaving 480 PLHIV for the cascade analysis.

**Table 1:** Sampled demographics of people living with HIV, Zambia 2018

	All PLHIV	Adults (age ≥ 15 years)	Children (age < 15 years)
**Total, n**	482	354	128
**Sex, n (weighted**^a^ **%)**			
Female	277 (54.6)	208 (54.9)	69 (46.3)
Male	205 (45.4)	146 (45.1)	59 (53.7)
**Median age in years (IQR), weighted**	33 (26–39)	33 (27–39)	4 (2–9)
**Age in years, n (weighted %)**			
< 2	30 (0.7)	–	30 (22.2)
2–4	39 (1.0)	–	39 (34.2)
5–9	28 (0.7)	–	28 (22.7)
10–14	31 (0.6)	–	31 (20.9)
15–24	60 (14.8)	60 (15.3)	–
25–34	131 (38.2)	131 (39.4)	–
35–44	115 (31.6)	115 (32.6)	–
45–54	39 (10.3)	39 (10.6)	–
55–64	7 (1.6)	7 (16.4)	–
≥ 65	2 (0.1)	2 (0.5)	–
**Province, n (weighted %)**			
Lusaka	321 (66.9)	234 (67.6)	87 (46.5)
Copperbelt	161 (33.1)	120 (32.4)	41 (53.5)
**Month of ART initiation, n (weighted %)**			
January	67 (12.4)	47 (12.1)	20 (18.9)
February	86 (17.5)	62 (17.6)	24 (13.7)
March	88 (19.5)	69 (19.7)	19 (13.4)
April	87 (15.8)	55 (15.6)	32 (24.4)
May	81 (17.4)	65 (17.6)	16 (12.0)
June	73 (17.4)	56 (17.4)	17 (17.6)

*Abbreviations*: *PLHIV* People living with HIV, *IQR* Interquartile range, *ART* Antiretroviral therapy

^a^Weighted for differential probabilities of selection across sampling clusters, age strata, and facilities

**Fig. 1:**
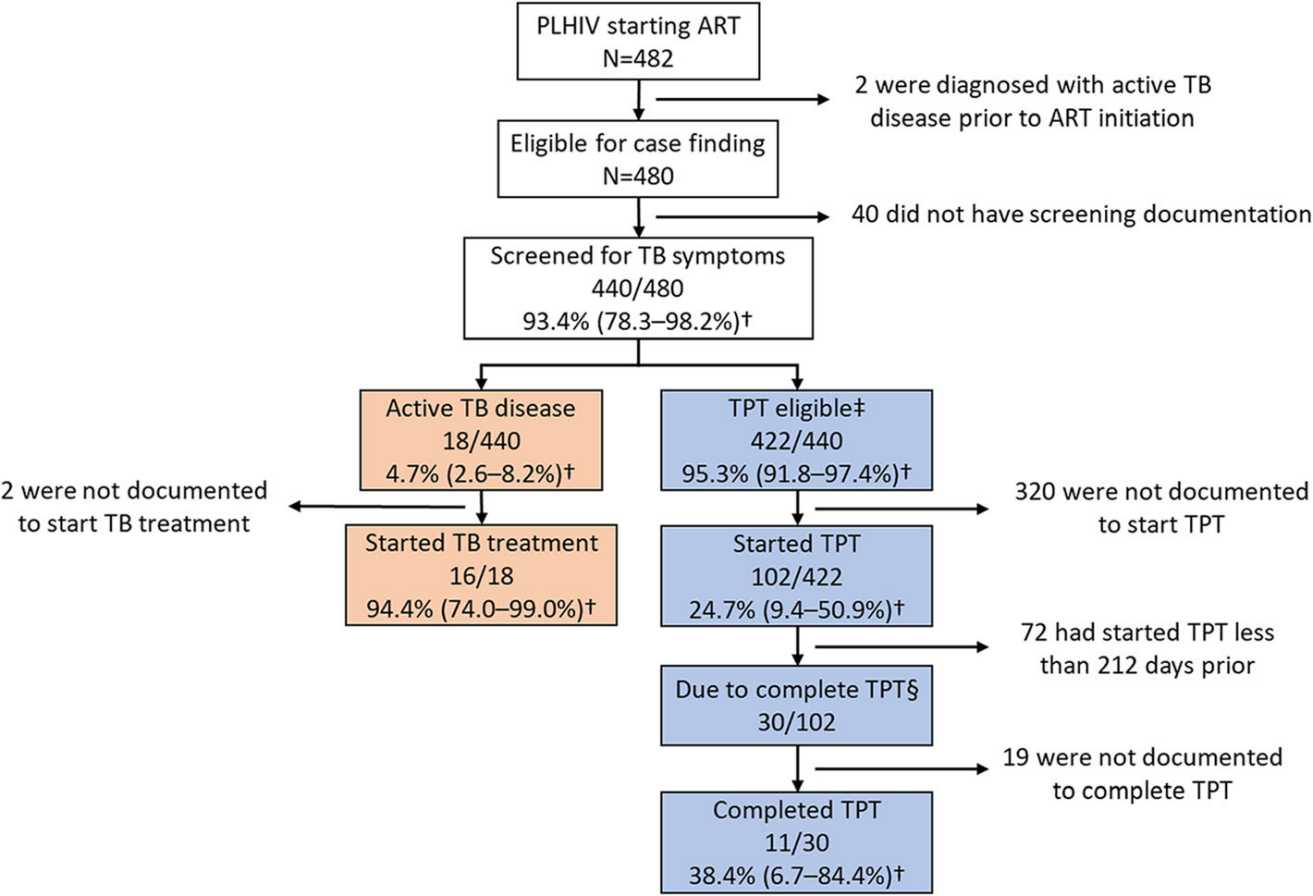
Counts, weighted percentages of people living with HIV through the tuberculosis case-finding/prevention cascade, Zambia 2018. Percentages are weighted for differential probabilities of selection across sampling clusters, age strata, and facilities. †95% confidence interval. ‡People living with HIV were considered TPT eligible if they were asymptomatic on TB screening and were not diagnosed with active TB disease, or symptomatic but were not diagnosed with active TB disease with ≥1 follow up appointment recorded. §People living with HIV were considered due to complete tuberculosis preventive treatment if > 189 days had elapsed since TPT initiation at the time of data abstraction. Abbreviations: PLHIV = people living with HIV, ART = antiretroviral therapy, TB = tuberculosis, TPT = tuberculosis preventive treatment

TB symptom screening was documented for 93.4% (95% CI: 78.3–98.2) of new-to-ART PLHIV (Fig. 1). Symptom screening was higher among adults (93.8%; 95% CI: 77.7–98.5%) compared with children (81.7%; 95% CI: 70.4–89.3%, *p* = 0.02) (Table 2). Among those screened for TB, 27.4% (95% CI: 17.3–40.4) had a positive symptom screen (i.e., had at least one of four symptoms). Adults were less likely to be symptomatic on screening (27.0%; 95% CI: 16.9–40.2%) than were children (42.3%; 29.6–56.2%, *p* = 0.01).

**Table 2:** Programmatic tuberculosis case finding and prevention indicators for people living with HIV, Zambia 2018

		Age strata		Province	
Indicator	All PLHIV, unweighted n/N, weighted^a^ % (95% CI)	Adults (≥15 years), unweighted n/N, weighted^a^ % (95% CI)	Children (< 15 years), unweighted n/N, weighted^a^ % (95% CI)	Lusaka, unweighted n/N, weighted^a^ % (95% CI)	Copperbelt, unweighted n/N, weighted^a^ % (95% CI)
1. Percentage of new-to-ART PLHIV who underwent TB symptom screening	440/480 93.4 (78.3–98.2)	334/353 **93.8**^e^ (77.7–98.5)	106/127 **81.7**^e^ (70.4–89.3)	291/321 91.6 (67.3–98.3)	149/159 96.9 (86.8–99.3)
2. Percentage of screened PLHIV who had a positive TB symptom screen (≥1 of 4 symptoms)	131/440 27.4 (17.3–40.4)	86/334 **27.0**^e^ (16.9–40.2)	45/106 **42.3**^e^ (29.6–56.2)	75/291 21.6 (12.5–34.8)	56/149 38.4 (16.1–67.0)
3. Percentage of PLHIV with a positive TB symptom screen who underwent microbiological evaluation^b^ for TB	41/131 51.9 (27.0–75.9)	36/86 **53.8**^e^ (27.7–78.0)	5/45 **7.0**^e^ (1.6–25.8)	17/75 43.0 (10.6–82.7)	24/56 61.6 (24.7–88.7)
4. Percentage of screened PLHIV who were diagnosed with active TB disease	18/440 4.7 (2.6–8.2)	17/334 **4.8**^e^ (2.7–8.3)	1/106 **0.3**^e^ (0.0–3.1)	10/291 4.2 (1.6–10.8)	8/149 5.6 (3.2–9.5)
5. Percentage of screened PLHIV who were TPT-eligible^c^	422/440 95.3 (91.8–97.4)	317/334 **95.2**^e^ (91.7–97.3)	105/106 **99.7**^e^ (96.9–100.0)	281/291 95.8 (89.2–98.4)	141/149 94.4 (90.5–96.8)
6. Percentage of TPT-eligible PLHIV who initiated TPT	102/422 24.7 (9.4–50.9)	89/317 **25.2**^e^ (9.6–51.7)	13/105 **7.7**^e^ (1.4–32.4)	87/281 **35.8**^e^ (15.0–63.8)	15/141 **3.1**^e^ (0.1–59.8)
7. Percentage of PLHIV who completed TPT among those due for completion^d^	11/30 38.4 (6.7–84.4)	9/24 38.5 (6.6–84.7)	2/6 26.6 (0.5–96.7)	11/26 40.0 (5.2–89.0)	0/4 0.0
8. Percentage who started and completed TPT among TPT-eligible PLHIV who started ART > 212 days prior to data abstraction	11/163 6.7 (1.0–34.0)	9/120 6.8 (1.0–34.8)	2/43 2.3 (0.4–11.1)	11/101 10.6 (1.4–50.3)	0/62 0.0

*Abbreviations*: *PLHIV* People living with HIV, *ART* Antiretroviral therapy, *TB* Tuberculosis, *CI* Confidence interval

^a^Weighted for differential probabilities of selection across sampling clusters, age strata, and facilities

^b^Microscopy, mycobacterial culture, or nucleic acid amplification test on a clinical specimen

^c^Screened for TB symptoms and asymptomatic and not diagnosed with active TB disease, or symptomatic and not diagnosed with active TB disease with ≥1 follow up appointment

^d^Started TPT > 212 days prior to data abstraction

^e^Statistically significant difference in indicator estimates across strata; Rao-Scott-adjusted chi-square *p* < 0.05

Among PLHIV with a positive symptom screen, 51.9% (95% CI: 27.0–75.9) underwent microbiological evaluation for active TB disease, of whom only 3.3% (95% CI: 0.3–24.5) had a documented positive result. Adults were more likely to undergo microbiological evaluation (53.8%; 95% CI: 27.7–78.0%) than were children (7.0%; 95% CI: 1.6–25.8%, *p* < 0.01) following a positive TB symptom screen. In total, 4.7% (95% CI: 2.6–8.2) of screened PLHIV were diagnosed with active TB disease (Fig. 1) with or without microbiologic confirmation, including 2.4% (95% CI: 0.7–7.7) of those with a negative symptom screen and 10.6% (95% CI: 7.0–15.7) of those with a positive symptom screen. Adults were more likely to be diagnosed with active TB disease (4.8%; 95% CI: 2.7–8.3%) than were children (0.3%; 95% CI: 0.0–3.1%, *p* < 0.01) (Table 2). TB treatment initiation was documented for 94.4% (95% CI: 74.0–99.0%) of PLHIV diagnosed with active TB disease (Fig. 1).

Overall, 95.3% (95% CI: 91.8–97.4) of screened PLHIV were TPT-eligible, including those who were asymptomatic, and those who were symptomatic but had at least one follow-up appointment and were not diagnosed with TB disease. Among TPT-eligible PLHIV, 24.7% (95% CI: 9.4–50.9) initiated TPT (Fig. 1), including 25.2% (95% CI: 9.6–51.7%) of adults versus 7.7% (95% CI: 1.4–32.4%) of children (*p* = 0.03) and 35.8% (95% CI: 15.0–63.8%) of PLHIV in Lusaka Province versus 3.1% (95% CI: 0.1–59.8%) of PLHIV in Copperbelt Province (*p* < 0.01) (Table 2). Notably, all TPT initiations in Copperbelt Province were from a single sampled ART facility; the other 3 Copperbelt facilities did not start TPT among any sampled PLHIV. In Lusaka Province, 2 of 8 facilities recorded no ART initiations among sampled PLHIV. Median date of TPT initiation was the same day as ART initiation (range 28 days before to 182 days after ART initiation). Limiting to PLHIV who were due for completion, (i.e., had started TPT > 212 days before data abstraction), 38.4% (95% CI: 6.7–84.4) completed TPT. Among all TPT-eligible PLHIV who started ART > 212 days prior to data abstraction, 6.7% (95% CI: 1.0–34.0) had started and completed TPT (Table 2).

### Healthcare worker interviews

We interviewed 13 HCWs at 12 ART facilities in Lusaka and Copperbelt Provinces including 9 (69.2%) nurses, 2 (15.4%) physicians, 1 (7.7%) clinical officer, and 1 (7.7%) pharmacist. Not all participants answered all questions.

Most (92.3%, 12/13) HCWs reported screening PLHIV for TB symptoms at each ART appointment (Table 3). However, 25.0% (3/12) respondents did not report using the full list of four WHO-recommended questions to screen for TB; two HCWs omitted night sweats and one omitted cough. Further, 41.7% (5/12) reported that a PLHIV must report ≥2 symptoms to screen positive and an additional 16.7% (2/12) reported this unless cough was included. For PLHIV with a positive symptom screen, all (12/12) respondents reported collecting a clinical specimen for microbiological evaluation.

**Table 3:** Healthcare worker interview responses, Zambia 2018

	Number of responses^a^, N (unweighted %)
Do you perform TB symptom screening at every ART appointment?	
Yes	12 (92.3)
No	1 (7.7)
Which symptoms do you use to screen for TB?	
Included 4 of 4 WHO-recommended screening symptoms for adults^b^	9 (75.0)
Omitted ≥1 of 4 symptoms	3 (25.0)
How many symptoms must a PLHIV have to screen positive?	
≥ 1	5 (41.7)
Cough alone is enough, otherwise ≥2	2 (16.7)
≥ 2	5 (41.7)
If a PLHIV has a positive symptom screen, do you collect sputum (or other specimen)?	
Yes	12
No	0
Do you receive TPT-specific training at your facility?	
Yes	3 (27.3)
No	8 (72.7)
Do you offer TPT to PLHIV without active TB disease?	
Yes	10 (83.3)
No	2 (16.7)
[If TPT is offered], do you reserve a 6-month supply of isoniazid for each patient?	
Yes	1 (16.7)
No	5 (83.3)
What is the most important barrier to providing TPT to PLHIV at your clinic?	
Isoniazid stockouts	5 (55.6)
Pyridoxine stockouts	2 (22.2)
Patients refuse TPT	1 (11.1)
Development of isoniazid-resistant *Mycobacterium tuberculosis*	1 (11.1)

*Abbreviations*: *PLHIV* Person/people living with HIV, *ART* Antiretroviral therapy, *TB* Tuberculosis, *TPT* TB Preventive treatment

^a^Not all respondents answered all questions

^b^Cough, fever, night sweats, weight loss

Only 27.3% (3/11) respondents reported receiving training on TPT at their facilities (Table 3). Although 83.3% (10/12) respondents reported offering TPT to PLHIV without active TB disease, 83.3% (5/6) reported an insufficient stockpile of isoniazid at the time of initiation for each patient to complete the 6-month course. One HCW who reported not offering TPT to eligible PLHIV said that the primary reason was an insufficient stockpile to ensure 6 months of treatment. Similarly, 55.6% (5/9) respondents reported isoniazid stockouts as the most important barrier to TPT provision. Two respondents (22.2%) reported pyridoxine stockouts and one respondent (11.1%) reported concern for promoting isoniazid-resistant strains of Mycobacterium tuberculosis as the primary reason for not prescribing TPT. Notably, at 3 of the 5 ART facilities which had not recorded any TPT initiations among sampled PLHIV, interviewed staff volunteered information that they did not start TPT while pyridoxine was stocked out, even if pyridoxine shortage was not cited as the most important barrier.

## Discussion

This survey revealed gaps throughout the TB case-finding and prevention cascade among PLHIV in Lusaka and Copperbelt Provinces. At each step from symptom screening to TPT completion and diagnosis of active TB disease, we noted deviations from national policies set by the MOH. However, this evaluation also informed multiple interventions enacted by the MOH aimed at increasing TB case-finding and improving TPT outcomes.

Gaps in TB case-finding began with deficiencies in TB screening. An estimated 6.6% of PLHIV did not undergo TB symptom screening at the time of ART initiation, including 18.3% of children, and, inconsistent with national guidelines [14], not all interviewed HCWs reported screening PLHIV at every visit. Further, 27.4% of those screened had at least one symptom, compared with approximately > 50% expected among ART-naïve adult PLHIV [9], suggesting gaps in quality as well as frequency of screening. Among interviewed HCWs, one quarter reported not using the full WHO-recommended symptom screen and more than half believed that the presence of a single symptom was insufficient for the PLHIV to screen positive. Although the sensitivity of the WHO-recommended symptom screen has been estimated near 90% among ART-naïve adult PLHIV [9], the effect of requiring more than one symptom for positivity or of omitting one or more symptom has not been evaluated. Consistent, evidence-based application of the WHO symptom screen could improve TB case detection among PLHIV and reduce the risk of ineffective TB treatment with isoniazid monotherapy.

This survey also revealed low utilization of TB diagnostic methods. Among PLHIV with a positive TB symptom screen, only half underwent microbiological evaluation for active TB disease, including < 10% of children. Poor access to TB diagnostics likely explains the low rate of microbiological evaluation overall; 5/12 sampled ART facilities did not have on-site TB diagnostic capabilities. For CLHIV in particular, TB diagnosis is further challenged by the difficulty in obtaining a clinical specimen [24], even in countries with sufficient access to diagnostic facilities. Consistent with this, children were more often symptomatic on TB screening, yet they were less likely to be diagnosed with active TB disease. Alternative diagnoses are myriad for children who present with cough, fever, and failure to gain weight. However, only one CLHIV was diagnosed with active TB disease in our sample, which may reflect underdiagnosis. Increasing local diagnostic capacity and improving the referral system for TB diagnosis may facilitate diagnosis of active TB disease among adults and children starting ART.

Even when active TB disease was not diagnosed, TPT was infrequently prescribed. Less than one quarter of eligible PLHIV started TPT, including < 10% of eligible children and < 5% of eligible PLHIV in Copperbelt Province. More than half of those who initiated were not documented to complete TPT. There are multiple possible reasons for low TPT uptake. Few interviewed HCWs had received training on TPT. Uncertainty about the benefits and risks of TPT for PLHIV may cause HCWs to deprioritize TPT relative to other clinical demands. One HCW, for example, cited concern for promoting mycobacterial resistance to isoniazid among patients with undiagnosed TB disease. To date, however, no study has demonstrated a significant increase in isoniazid resistance following trials of TPT [20, 25]. HCWs may also underestimate the benefits and overestimate the risks of TPT in CLHIV, who tolerate isoniazid with fewer adverse effects than adults, and are more likely to progress to active TB disease if infected [26]. Provider training on TPT risks and benefits has led to increases TPT prescription for PLHIV in resource limited settings [20].

However, supply chain gaps were the most common barrier to TPT cited by HCWs, with many providers reporting insufficient stockpiles of isoniazid for initiated patients to complete 6 months. Pediatric-friendly formulations with adjustable weight-based dosing posed an additional procurement challenge. Although TPT demand was largely driven by the HIV program, isoniazid procurement was managed by the National TB Program (NTP). Requisition quantities were driven by the number of cases of TB disease reported by facility, rather than the number of PLHIV accessing care [27]. Finally, even when isoniazid was available, pyridoxine was often not, due to disjointed procurement protocols and differing quantities ordered. Pyridoxine shortage was reported as a contributing factor against TPT initiation at several ART facilities which had not recorded any TPT initiations among sampled PLHIV.

In late 2019, recognizing these supply-chain inefficiencies, the MOH, with PEPFAR support, began procuring isoniazid (adult and pediatric dosages) and pyridoxine in tandem. To address concerns regarding stockouts, the NTP simultaneously modified forecasting and distribution procedures, linking isoniazid supply to number of PLHIV served. High-volume facilities began to receive full six-month treatment courses of isoniazid with pyridoxine for all eligible PLHIV in the facility, enabling rapid scale-up without fear of shortage.

Additionally, in March 2019 the NTP released updated guidelines for the management of latent TB infection, expanding TPT eligibility to PLHIV already on ART [28]. Through this initiative and changes in supply chain, the number of PLHIV completing TPT through PEPFAR support in Zambia tripled from 2018 to 2019 [13]. Continuing aggressive TPT scale-up, in 2020 the MOH implemented a TPT surge campaign aimed at initiating 100,000 PLHIV on TPT in each of three 60-day phases, ultimately achieving an additional four-fold increase in annual TPT completions. As part of this campaign, providers received enhanced training on TB symptom screening, diagnostic referral, and TPT initiation and management. Local TB diagnostic capacity was also increased through introduction of point-of-care testing for urine lipoarabinomannan antigen.

This evaluation had at least five limitations. First, incomplete data recording in medical records may have led to underestimation of some programmatic indicators. Second, we did not sample facilities outside of Lusaka and Copperbelt Provinces and findings may not be generalizable to all ART facilities in Zambia. However, these two provinces were selected because they carry the highest national burden of TB and HIV. Improving TPT uptake in these provinces will be crucial in reducing national TB mortality among PLHIV. Third, available follow up times for sampled PLHIV ranged from 3 to 9 months, depending on the date of ART initiation relative to the date of data abstraction; some PLHIV may have started TPT after data abstraction. Fourth, the assessment was underpowered to detect statistically significant differences in indicators across demographic and geographic strata. Nonetheless, there were some marked differences noted which can be informative for programmatic intervention. Finally, interviewed HCWs were sampled by convenience, possibly introducing selection bias. Not all respondents answered all interview questions, possibly introducing response bias toward answers perceived to be acceptable to the interviewers. However, including HCW perspectives allowed us to better understand barriers and informed key programmatic interventions enacted by the MOH.

## Conclusions

This survey was the first to quantify losses at each stage of the TB case-finding and prevention cascade among PLHIV in Zambia. Less than 25% of those eligible started TPT and < 10% completed TPT within 6 months of starting ART. The reasons included inconsistent implementation of the WHO-recommended TB symptom screen, low access to TB diagnostics, unmet demand for TPT training among HCWs, and supply chain inefficiencies. The MOH has begun to address some of these shortfalls through intensified provider training on TB screening/diagnosis and TPT management, coordinated isoniazid and pyridoxine procurement, and forecasting procurements according to estimated HIV disease burden. These strategies will be crucial for continued TPT scale-up in Zambia and to meaningfully reduce HIV- and TB-associated mortality.

## Supplementary Information

**Additional file 1: Figure S1.** Sampling diagram demonstrating stratified two-stage cluster sampling. To achieve a representative sample of people living with HIV, we first sampled facilities providing antiretroviral therapy with probability-proportional-to-size; in this example, Facilities C, D, and F are sampled from a sampling frame of six facilities (A). At each sampled facility, we systematically sampled 30 adults and 30 children from a registry; in this example with *N* = 210 adult patients in Facility F, a sampling interval of N/30 = 7 is used to sample 30 adult patients for inclusion in the survey (B).

## Data Availability

The datasets used and analyzed during the current study are available from the corresponding author on reasonable request.

## Abbreviations

ART: Antiretroviral therapy
CI: Confidence interval
CLHIV: Children living with human immunodeficiency virus
HCW: Healthcare worker
HIV: Human immunodeficiency virus
IQR: Interquartile range
LTBI: Latent tuberculosis infection
MOH: Ministry of Health (Zambia)
NTP: National Tuberculosis Program (Zambia)
PEPFAR: United States President’s Emergency Plan for AIDS Relief
PLHIV: People living with human immunodeficiency virus
TB: Tuberculosis
TPT: Tuberculosis preventive treatment
WHO: World Health Organization

## References

[1] World Health Organization. Global tuberculosis report, 2020. Geneva: WHO; 2020. License: CC BY-NC-SA 3.0 IGO. https://www.who.int/publications/i/item/9789240013131. Accessed Apr 2021

[2] Suthar AB, Lawn SD, del Amo J, Getahun H, Dye C, Sculier D, Sterling TR, Chaisson RE, Williams BG, Harries AD, Granich RM (2012). Antiretroviral therapy for prevention of tuberculosis in adults with HIV: a systematic review and meta-analysis. PLoS Med.

[3] Dye C, Williams B (2019). Tuberculosis decline in populations affected by HIV: a retrospective study of 12 countries in the WHO African region. Bull World Health Organ.

[4] Surie D, Borgdorff MW, Cain KP, Click ES, DeCock KM, Yuen CM (2018). Assessing the impact of antiretroviral therapy on tuberculosis notification rates among people with HIV: a descriptive analysis of 23 countries in sub-Saharan Africa, 2010–2015. BMC Infect Dis.

[5] Rangaka MX, Wilkinson RJ, Boulle A, Glynn JR, Fielding K, van Cutsem G, Wilkinson KA, Goliath R, Mathee S, Goemaere E, Maartens G (2014). Isoniazid plus antiretroviral therapy to prevent tuberculosis: a randomized, double-blind, placebo-controlled trial. Lancet.

[6] Danel C, Moh R, Gabillard D, Badje A, le Carrou J, Ouassa T (2015). The TEMPRANO ANRS 12136 study group. A trial of early antiretrovirals and isoniazid preventive therapy in Africa. N Engl J Med.

[7] Badje A, Moh R, Gabillard D, Kabran M, Ntakpe JB, Carrou JL (2017). Effect of isoniazid preventive therapy on risk of death in west African, HIV-infected adults with high CD4 cell counts: long term follow-up of the TEMPRANO ANRS 12136 trial. Lancet Glob Health.

[8] World Health Organization (2018). Latent tuberculosis infection: updated and consolidated guidance for programmatic management. WHO/CDS/TB/2018.4.

[9] Hamada Y, Lujan J, Schenkel K, Ford N, Getahun H (2018). Sensitivity and specificity of WHO’s recommended four-symptom screening rule for TB in PLHIV: a systematic review and meta-analysis. Lancet HIV.

[10] Kapata N, Chanda-Kapata P, Ngosa W, Metitiri M, Klinkenberg E, Kalisvaart N, Sunkutu V, Shibemba A, Chabala C, Chongwe G, Tembo M, Mulenga L, Mbulo G, Katemangwe P, Sakala S, Chizema-Kawesha E, Masiye F, Sinyangwe G, Onozaki I, Mwaba P, Chikamata D, Zumla A, Grobusch MP (2016). The prevalence of tuberculosis in Zambia: results from the first national TB prevalence survey, 2013–2014. PLoS One.

[11] Ministry of Health Zambia (2019). Zambia population based HIV impact assessment (ZAMPHIA) 2016: final report.

[12] Joint United Nations Programme on HIV/AIDS (2020). UNAIDS Data 2020.

[13] The United States President’s Emergency Plan for AIDS Relief (2021). PEPFAR panorama spotlight.

[14] Ministry of Health Zambia (2018). Zambia consolidated guidelines for prevention and treatment of HIV infection.

[15] Teklay G, Teklu T, Legesse B, Tedla K, Klinkenberg E (2016). Barriers in the implementation of isoniazid preventive therapy for people living with HIV in northern Ethiopia: a mixed quantitative and qualitative study. BMC Public Health.

[16] Surie D, Interrante JD, Pathmanathan I, Patel MR, Anyalechi G, Cavanaugh JS, Kirking HL (2019). Policies, practices and barriers to implementing tuberculosis preventive treatment—35 countries, 2017. Int J Tuberc Lung Dis.

[17] Getahun H, Granich R, Sculier D, Gunneberg C, Blanc L, Nunn P, Raviglione M (2010). Implementation of isoniazid preventative therapy for PLHIV worldwide: barriers and solutions. AIDS.

[18] Ayele HT, van Mourik MS, Bonten MJ (2016). Predictors of adherence to isoniazid preventive therapy in people living with HIV in Ethiopia. Int J Tuberc Lung Dis.

[19] Maharaj B, Gengiah TN, Yende-Zuma N, Gengiah S, Naidoo A, Naidoo K (2017). Implementing isoniazid preventive therapy in a tuberculosis treatment-experienced cohort on ART. Int J Tuberc Lung Dis.

[20] Pathmanathan I, Ahmedov S, Pevzner E, Anyalechi G, Modi S, Kirking H, Cavanaugh JS (2018). TB preventive therapy for people living with HIV: key considerations for scale-up in resource-limited settings. Int J Tuberc Lung Dis.

[21] World Health Organization (2018). Global tuberculosis report, 2018. WHO/CDS/TB/2018.20.

[22] Coffman J, Chanda-Kapata P, Marais BJ, Kapata N, Zumla A, Negin J (2017). Tuberculosis among older adults in Zambia: burden and characteristics among a neglected group. BMC Public Health.

[23] PASS: Power Analysis and Sample Size Software (2017). Version 15.0.4.

[24] Khan EA, Starke JR (1995). Diagnosis of tuberculosis in children: increased need for better methods. Emerg Infect Dis.

[25] van Halsema CL, Fielding KL, Chihota VN, Russell EC, Lewis JJC, Churchyard GJ, Grant AD (2010). Tuberculosis outcomes and drug susceptibility in individuals exposed to isoniazid preventive therapy in a high HIV prevalence setting. AIDS.

[26] Getahun H, Matteelli A, Chaisson RE, Raviglione M (2015). Latent mycobacterium tuberculosis infection. N Engl J Med.

[27] Kagujje M, Mubiana ML, Mwamba E, Muyoyeta M (2019). Implementation of isoniazid preventive therapy in people living with HIV in Zambia: challenges and lessons. BMC Public Health.

[28] Ministry of Health Zambia (2019). Guidelines for the Management of Latent Tuberculosis Infection.

