# Supplementary material for: Assessment of the tuberculosis case-finding and prevention cascade among people living with HIV in Zambia – 2018: a cross-sectional cluster survey
Source: BMC Public Health. 2021 May 4;21:859. doi: 10.1186/s12889-021-10929-z (PMC8094475; doi:10.1186/s12889-021-10929-z)
Supplement: Supplementary file 1 — Additional file 1: Figure S1. Sampling diagram demonstrating stratified two-stage cluster sampling. To achieve a representative sample of people living with HIV, we first sampled facilities providing antiretroviral therapy with probability-proportional-to-size; in this example, Facilities C, D, and F are sampled from a sampling frame of six facilities (A). At each sampled facility, we systematically sampled 30 adults and 30 children from a registry; in this example with N = 210 adult patients in Facility F, a sampling interval of N/30 = 7 is used to sample 30 adult patients for inclusion in the survey (B). [file 12889_2021_10929_MOESM1_ESM.docx]

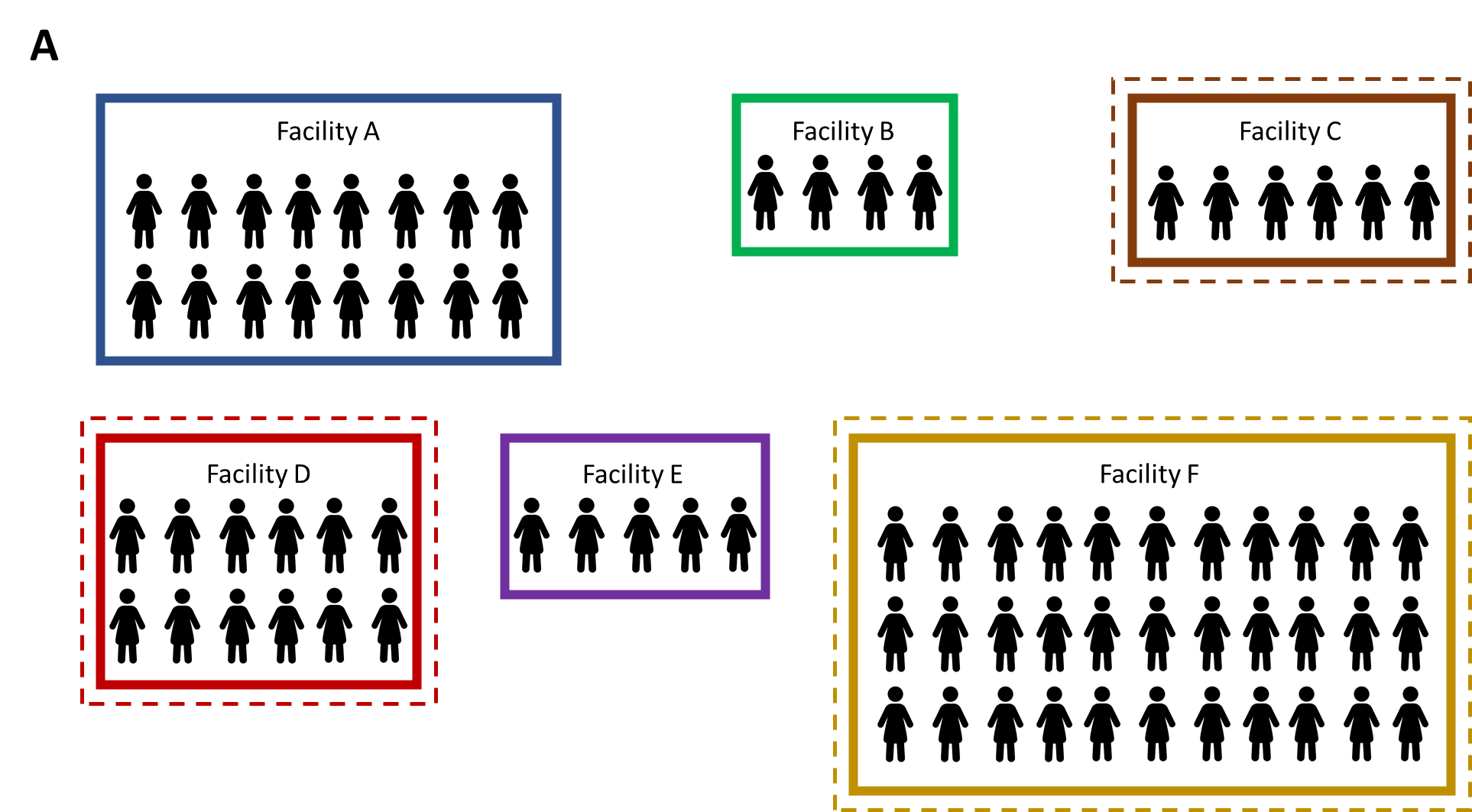


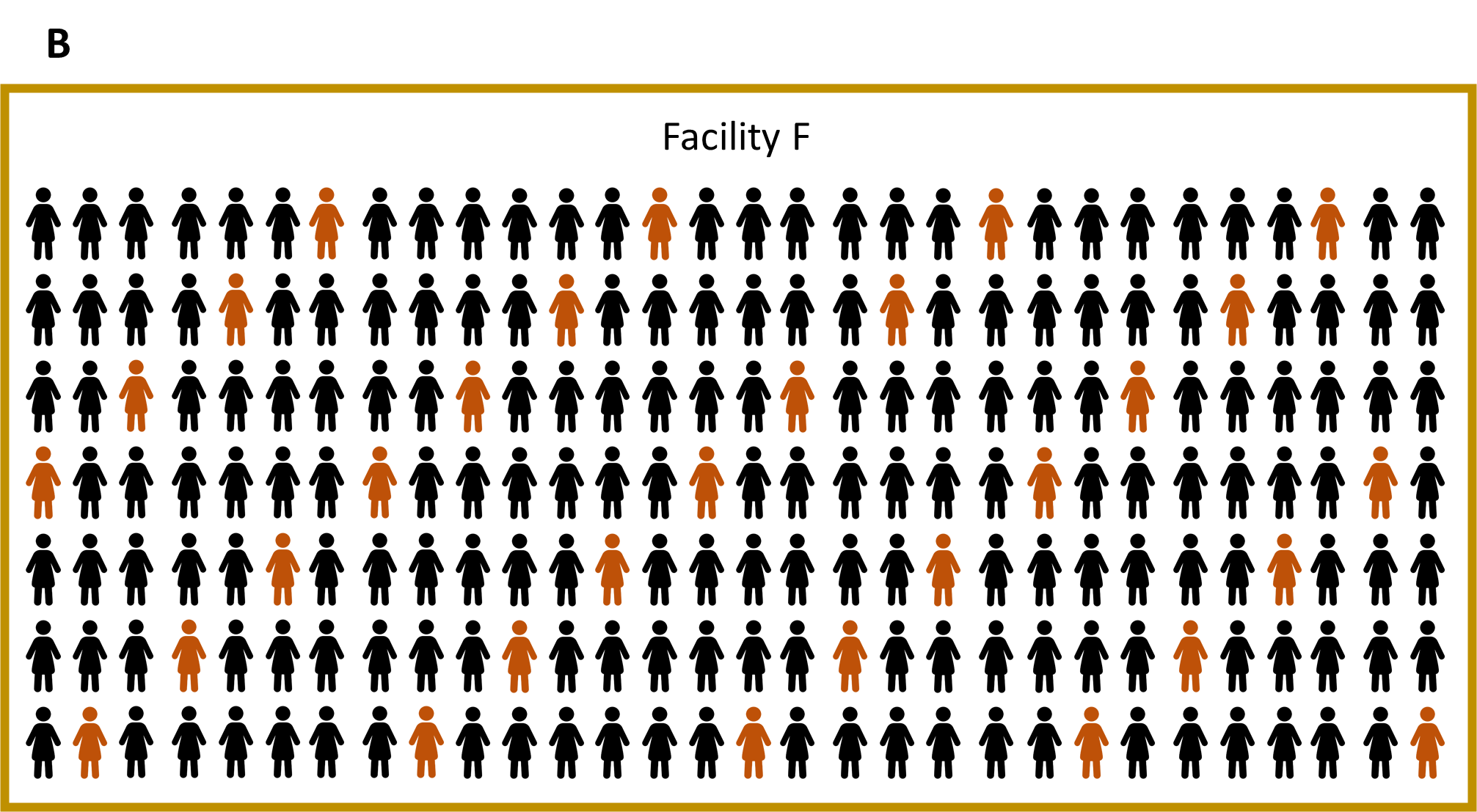


**Supplemental Figure. Sampling diagram demonstrating stratified two-stage cluster sampling.** To achieve a representative sample of people living with HIV, we first sampled facilities providing antiretroviral therapy with probability-proportional-to-size; in this example, Facilities C, D, and F are sampled from a sampling frame of six facilities (A). At each sampled facility, we systematically sampled 30 adults and 30 children from a registry; in this example with N=210 adult patients in Facility F, a sampling interval of N/30=7 is used to sample 30 adult patients for inclusion in the survey (B).
